# Adaptación transcultural para Colombia y validez de contenido de la escala RAC de evaluación del riesgo de infección en el adulto hospitalizado**

**DOI:** 10.15649/cuidarte.2406

**Published:** 2022-08-28

**Authors:** Alba Luz Rodríguez-Acelas, Mónica López de Ávila, Daniela Yampuezán Getial, Miriam de Abreu Almeida, Wilson Cañon-Montañez

**Affiliations:** 1 Facultad de Enfermería, Universidad de Antioquia, Medellín, Colombia. E-mail: aluz.rodriguez@udea.edu.co Universidad de Antioquia Facultad de Enfermería Universidad de Antioquia Medellín Colombia aluz.rodriguez@udea.edu.co; 2 Facultad de Enfermería, Universidad de Antioquia, Medellín, Colombia. E-mail: monica.lopezd@udea.edu.co Universidad de Antioquia Facultad de Enfermería Universidad de Antioquia Medellín Colombia monica.lopezd@udea.edu.co; 3 Facultad de Enfermería, Universidad de Antioquia, Medellín, Colombia. E-mail: daniela.yampuezang@udea.edu.co Universidad de Antioquia Facultad de Enfermería Universidad de Antioquia Medellín Colombia daniela.yampuezang@udea.edu.co; 4 Escola de Enfermagem, Universidade Federal do Rio Grande do Sul, Porto Alegre, Brasil. E-mail: miriam.abreu2@gmail.com Universidade Federal do Rio Grande do Sul Escola de Enfermagem Universidade Federal do Rio Grande do Sul Porto Alegre Brazil miriam.abreu2@gmail.com; 5 Facultad de Enfermería, Universidad de Antioquia, Medellín, Colombia. E-mail: wilson.canon@udea.edu.co Universidad de Antioquia Facultad de Enfermería Universidad de Antioquia Medellín Colombia wilson.canon@udea.edu.co

**Keywords:** Medición de Riesgo, Control de Infecciones, Comparación Transcultural, Estudio de Validación, Seguridad del Paciente, Risk Assessment, Infection Control, Cross-Cultural Comparison, Validation Study, Patient Safety, Medido de Risco, Controle de Infecgóes, Comparado Transcultural, Estudo de Validado, Seguranza do Paciente

## Abstract

**Introducción:**

Las Infecciones Asociadas a la Atención en Salud (IAAS) son un grave problema de salud pública, que puede ser prevenidas al identificar los factores de riesgo con el uso de escalas. Objetivo: Adaptar transculturalmente y realizar la validación de contenido y de face de la escala Rodríguez-Almeida-Cañon (RAC) de evaluación del riesgo de infección en adultos hospitalizados.

**Materiales y Métodos:**

Estudio metodológico de adaptación transcultural. La recolección de datos se realizó de junio a noviembre de 2020. La muestra estuvo compuesta por 11 especialistas. La escala RAC se evaluó en su conjunto, determinando su alcance, los ítems fueron evaluados individualmente, verificando su claridad, relevancia y pertinencia. Para evaluar cada ítem se utilizó una escala tipo *Likert* de cuatro niveles. La validez de contenido fue evaluada a través del índice de validez de contenido (IVC).

**Resultados:**

Por medio de la evaluación del comité de especialistas fue posible determinar que la escala RAC es apta para uso en el contexto cultural colombiano. Se realizaron ajustes para mejorar la interpretación de algunos ítems. El IVC de los ítems estuvo entre 0.90 a 1.0 y el IVC promedio de la escala fue de 0.98.

**Discusión:**

Esta escala permite medir el riesgo de IAAS a un bajo costo, con el fin de poder planear y ejecutar intervenciones por parte del equipo multidisciplinario que tiene a cargo la salud y el cuidado del paciente.

**Conclusiones:**

La escala RAC en su versión en español es un instrumento apropiado para la evaluación del riesgo de IAAS en el adulto hospitalizado en Colombia.

## Abstract (en)

**Introduction:**

Health care-associated infections (HAI) are a serious public health problem, which can be prevented by identifying risk factors with the use of scales. Objective: To adapt cross-culturally and perform content and face validation of the Rodríguez-Almeida-Cañon (RAC) scale for assessing the risk of infection in hospitalized adults.

**Materials and Methods:**

Methodological study of cross-cultural adaptation. Data collection was carried out from June to November 2020. The sample consisted of 11 specialists. The RAC scale was evaluated as a whole, determining its scope, the items were evaluated individually, verifying their clarity, relevance and pertinence. To evaluate each item, a four-level Likert-type scale was used. The content validity was evaluated through the content validity index (CVI).

**Results:**

Through the evaluation of the committee of specialists it was possible to determine that the RAC scale is suitable for use in the Colombian cultural context. Adjustments were made to improve the interpretation of some items. The CVI of the items was between 0.90 to 1.0 and the average CVI of the scale was 0.98.

**Discussion:**

This scale makes it possible to measure the HAI risk at a low cost, in order to be able to plan and execute interventions by the multidisciplinary team in charge of the health and care of the patient.

**Conclusions:**

The RAC scale in its Spanish version is an appropriate instrument for assessing the risk of HAI in hospitalized adults in Colombia.

## Resumo

**Introdujo:**

As infecgóes associadas a assistencia a saúde (IAAS) sao um grave problema de saúde pública, que pode ser prevenido por meio da identificado de fatores de risco com o uso de escalas. Objetivo: Adaptar transculturalmente e realizar a validado de conteúdo e de face da escala Rodríguez-Almeida-Cañon (RAC), de avaliagáo do risco de infecgáo em adultos hospitalizados.

**Materiais e Métodos:**

Estudo metodológico de adaptado transcultural. A coleta de dados foi realizada no período de junho a novembro de 2020. A amostra foi composta por 11 especialistas. A escala RAC foi avaliada como um todo, determinando seu escopo, os itens foram avaliados individualmente, verificando sua clareza, relevancia e pertinencia. Para avaliar cada item, foi utilizada uma escala do tipo Likert de quatro níveis. A validade de conteúdo foi avaliada por meio do índice de validade de conteúdo (IVC).

**Resultados:**

Por meio da avaliagáo do comite de especialistas, foi possível constatar que a escala RAC é adequada para uso no contexto cultural colombiano. Ajustes foram feitos para melhorar a interpretado de alguns itens. O IVC dos itens ficou entre 0,90 a 1,0 e o IVC médio da escala foi de 0,98.

**Discussao:**

Esta escala permite mensurar o risco de IAAS a baixo custo, de forma a poder planejar e executar intervengóes da equipe multiprofissional responsável pela saúde e cuidado do paciente.

**Condusdes:**

A escala RAC em sua versáo em espanhol é um instrumento adequado para a avaliagáo do risco de IAAS em adultos hospitalizados na Colombia.

## Introducción

Las Infecciones Asociadas a la Atención en Salud (IAAS) son consideradas un problema de salud pública, según la Organización Mundial de la Salud (OMS) la prevalencia de adquirir una IAAS en países en desarrollo, es el doble en comparación con los países desarrollados, con una prevalencia entre 3.5 al 12%; sin embargo, en países en desarrollo la prevalencia oscila alrededor del 25%^1^, generando una gran repercusión en el estado de salud de los pacientes, la familia y la comunidad^2^, esto se ve reflejado en el aumento de los días de hospitalización, en la demanda del tratamiento, procedimientos clínicos e incluso la morbimortalidad, además del aumento en los costos para el sistema de salud y asimismo, todo el sufrimiento, dolor y deterioro en la calidad de vida de las personas y familia^3^.

De acuerdo con la OMS y el Centro para el Control y Prevención de Enfermedades (CDC), en el año 2016 el costo anual atribuido a las IAAS osciló entre 35.7 y 45 mil millones de dólares en Estados Unidos, mientras que en Europa ese impacto económico ascendió a 7000 millones de euros^4^. En América Latina no se conoce un dato exacto de la carga de enfermedad que genera estas infecciones, debido a que no todos los países cuentan con un sistema de vigilancia efectivo y constante, pero se ha demostrado que las IAAS son un factor importante de morbilidad y mortalidad^5^.

Con respecto a Colombia, durante el 2020 se reportaron 6306 casos solo de infecciones asociadas a dispositivos en Unidad de Cuidados Intensivos (UCI), reflejando un aumento del 23.6% comparado con el período 2019^6^. Para el 2018, en el departamento de Antioquia se reportó una tasa de prevalencia de infección del torrente sanguíneo asociado a catéter de 1.5 casos por 1000 días de catéter central, 1.6 casos por 1000 días de uso de sonda vesical para infección sintomática del tracto urinario asociada a catéter y 2.3 por cada 1000 días de ventilador por neumonía asociada a ventilación mecánica^7^.

Según la evidencia, las IAAS pueden ser consideradas un evento prevenible y basado en el Protocolo de Londres^8^, se ha identificado que las causas de las IAAS pueden estar relacionadas con la sobrecarga de los profesionales de la salud, políticas institucionales poco claras, deficiencia en los programas de inducción, falta de implementación de rondas de seguridad, inadecuada higiene de manos en los cinco momentos y/o inapropiada manipulación de los instrumentos médicos, entre otros.

Una de las herramientas que contribuye a la identificación de dichos factores, son las escalas de evaluación de riesgo, las cuales surgen a partir de evidencias científicas y la Práctica Basada en la Evidencia^9^, este tipo de herramientas diagnósticas deben pasar por un proceso riguroso de construcción y validación demostrando su capacidad de medir el evento en estudio^10^, una vez que sea eficaz y se adapte a los diferentes contextos culturales^11^; otro aspecto considerado es la optimización de tiempo invertido en su aplicación, dando respuesta al fenómeno en medición^12^. En la actualidad existe una gran diversidad de escalas de evaluación para uso hospitalario que buscan medir el riesgo de infección, como la *Central Line Associated Blood Stream Infections* (CLABSI), que busca medir el riesgo del catéter central insertado periféricamente^13^. También se encuentra el *Infection Risk Scan* (IRIS), que surgió a partir del número elevado de casos por IAAS, esta herramienta busca generar un control de infecciones mediante la identificación de factores riesgo propios del paciente, servicio y atención, con el fin de que sean intervenidos y evaluados continuamente, para realizar seguimiento al ciclo de control de calidad^14^.Muchos de los instrumentos existentes han sido de uso limitado, debido a interferencias culturales, falta de procesos de validación, etc.; con todo, su uso va a depender de que se realicen Adaptaciones Transculturales (AT), para que se adapten a diversos contextos culturales e idiomas; así mismo, el empleo de herramientas transversales va a permitir la socialización de conocimientos, resultados, intervenciones y soluciones a experiencias de los profesionales de la salud en el ámbito clínico y en las diferentes regiones del mundo^11^.

Con la AT se busca generar acceso a instrumentos ya existentes y validados, por medio de un proceso de traducción que se adapte al contexto cultural de determinadas regiones, manteniendo correspondencia con el instrumento original a través de la equivalencia de contenido, equivalencia semántica, equivalencia técnica, equivalencia de criterio y equivalencia conceptual^11^, buscando conservar la congruencia con el instrumento original^15^. En este proceso participan además de traductores, un comité de especialistas que cumplan criterios de conocimientos y experiencia en el tema, con el fin de mantener el carácter evaluativo^11^.

Dado el apoyo científico-práctico que generan las escalas para el contexto clínico y el limitado proceso de AT de los diferentes instrumentos evaluativos, se encontró que en Colombia no existe un instrumento que mida el riesgo de IAAS; sin embargo, existe la *Escala de Avaliagáo do Risco para Infecido no Adulto Hospitalizado*^*16*^*,* creada en Brasil, la cual fue desarrollada a partir de una Revisión Sistemática y Metaanálisis^17^, seguida de una validación de contenido por 23 especialistas que resultó en un Índice de Validez de Contenido (IVC) de los ítems entre 0.83 y 1.0^18^. Posteriormente se realizó la evaluación de las propiedades psicométricas y confiabilidad de la escala a través de un estudio de cohorte prospectivo^16^, la cual generó un instrumento compuesto por un modelo bidimensional constituido por dos factores: intrínsecos con 8 ítems, definido como aquellas condiciones propias del paciente al momento del ingreso y extrínsecos con 7 ítems, entendido como los factores que involucran el tratamiento que se la ha dado al paciente^19^, este rigor metodológico permitió concluir en una escala válida y confiable para su aplicación en el medio brasilero; por consiguiente, se estableció este estudio que tiene como objetivo realizar la adaptación transcultural y evaluar la validez de contenido al español de la Escala RAC de Evaluación del Riesgo de Infección en el adulto hospitalizado para uso en Colombia.

## Materiales y Métodos

### Diseño del estudio

**Figura 1:**
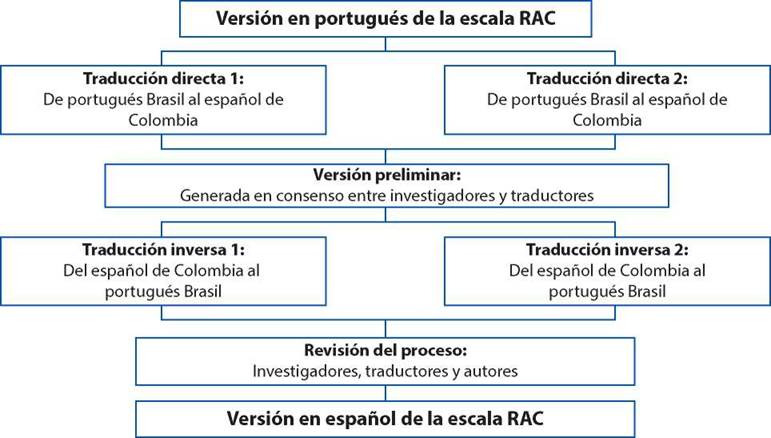
Proceso de adaptación transcultural y validez de contenido de la escala RAC

Es un estudio metodológico de AT y estimación de la validez de contenido de la Escala RAC de Evaluación del Riesgo de Infección en el Adulto Hospitalizado al español de Colombia, teniendo en cuenta los procedimientos psicométricos recomendados en la literatura^20^. Para ello, se llevaron a cabo los pasos sugeridos por la *Guidelines for the Process of Cross-Cultural Adaptation of Self-Report Measures*^21^ que incluye las siguientes etapas: traducción inicial del instrumento; síntesis de traducciones; traducción inversa y evaluación por parte de especialistas y revisión del proceso. Los pasos que involucran a los traductores se detallan en la Figura 1.

### Traducción, retro-traducción y síntesis

Para la etapa inicial de traducción, se tuvieron en cuenta dos traductores bilingües independientes procedentes de Colombia, pero con amplio dominio del idioma portugués brasilero, los cuales realizaron las traducciones, buscando obtener una traducción lo más coherente y puntual con el español colombiano, estos traductores desconocían el fin del estudio para evitar cualquier tipo de sesgo. De este primer paso, se derivaron las dos primeras versiones de la escala: Versión Español 1 y Versión Español 2. Posteriormente se realizó la síntesis de las traducciones, entre investigadores, autores y traductores, donde se verificaron, compararon y consensuaron las discrepancias para lograr establecer la Síntesis de la Versión de español (SVE).

En el siguiente paso, correspondiente a la retro-traducción, se envió la SVE a dos traductores bilingües brasileros, con dominio lingüístico del español colombiano, quienes también desconocían el objetivo del estudio, este paso se realizó con el fin de verificar las diferencias conceptuales o semánticas y comprobar que la escala mantenía su mismo contenido al ser retro-traducida. Las versiones de retro-traducción 1 y 2 fueron igualmente revisadas en conjunto con el equipo de traductores e investigadores, buscando comprobar la similitud entre ambas y discutiendo las diferencias hasta que se logró un consenso.

Finalmente, se presentó a los traductores e investigadores la versión traducida y comparada con la versión original, posibilitando confrontar las versiones y la realización de ajustes en cuanto a significados e interpretaciones durante el proceso, obteniendo de esta manera, la Versión Final en Español (VFE) de la escala RAC.

### Validación de contenido por especialistas

Una vez obtenida la VFE, se procedió con la conformación del comité de especialistas para llevar a cabo la revisión de la versión final traducida, donde se evaluó la comprensión y claridad del instrumento, la forma y la equivalencia semántica, conceptual y cultural del mismo^22^. Para llevar a cabo la puntuación, se empleó la escala de *Likert* teniendo en cuenta tres calificaciones: 1- Totalmente en desacuerdo, 2- Parcialmente de acuerdo, 3- Totalmente de acuerdo. Posteriormente, se verificó que las particularidades de cada ítem de la escala original se conservarán durante todo el proceso de adaptación transcultural.

### Tipo de muestreo y criterios de elegibilidad de especialistas

Para la etapa de validación de contenido por especialistas, los participantes fueron seleccionados por muestreo de efecto bola de nieve, cumpliendo al menos dos de los siguientes criterios: título de maestría y/o doctorado; experiencia mínima de dos años en unidades de hospitalización para adultos, experiencia mínima de un año en el Comité de Control de Infecciones Hospitalarias, y experiencia con investigaciones y/o publicaciones sobre IAAS, quedando conformado un grupo de 11 profesionales de la salud.

Una vez identificados, se contactaron por medio de correo electrónico y aceptaron la invitación, se les envió el enlace de un formulario de *Google* con las instrucciones que se requieren para el entendimiento del estudio, el límite de tiempo para la devolución del formulario diligenciado fue un mes, se convocó un número superior previendo aquellos que no respondieran, los cuales fueron excluidos.

La validación de contenido fue evaluada por medio del Índice de Validez de Contenido (IVC), se determinó la proporción con la que los especialistas estuvieron de acuerdo con las características del cuestionario y cada uno de sus ítems, en cuanto a relevancia teórica, claridad y pertinencia práctica. Cada uno de los ítems de la escala, fueron evaluados a través de la media de la I-IVC, calculados por separados y divididos en el número de ítems considerados en la evaluación. De igual manera, de acuerdo con la literatura, el criterio de aceptación entre el comité de especialistas para la validación de cada ítem se fijó en un puntaje de concordancia superior a 0.80^20^^,^^23^.

### Tabulación y análisis estadístico de los datos

Todos los datos que se recolectaron en el estudio fueron organizados en una hoja de cálculo en Excel y exportados para análisis estadístico en el programa Stata v.16.0. La base de datos fue almacenada en Mendeley Data^24^. Se tuvo en cuenta la caracterización de los especialistas, la cual incluye sexo, departamento de residencia, estado civil, titulación académica, edad, experiencia, dentro de la evaluación de la escala se incluyeron parámetros como claridad, pertinencia y relevancia teórica de cada uno de los ítems. Las variables categóricas fueron presentadas mediante frecuencias absolutas y relativas. Las variables continuas se presentan con el promedio y desviación estándar. Para evaluar la validez de contenido, se calculó el IVC para cada ítem, utilizando la siguiente formula: IVC= (número de respuesta con una puntuación de 2 ó 3) / (número total de respuestas)^25^. ***-***

### Consideraciones éticas

Este estudio se clasifica sin riesgo, según las Directrices y Normas Reguladoras de Investigaciones que involucra a Seres Humanos, de acuerdo con la Resolución 008430 de 1993 del Ministerio de Salud de Colombia^26^, también se contemplaron los principios éticos de Ezekiel Emanuel para la investigación clínica^27^, la aplicación del consentimiento informado por parte de los especialistas y aprobación del Comité de Ética institucional N° 2018-22470.

## Resultados

Los resultados obtenidos durante el estudio abarcan el proceso de AT y Validación de contenido, buscando obtener una escala para uso en el contexto cultural colombiano.

### Traducción, retro-traducción y síntesis

En el proceso de traducción de la escala original en portugués brasilero a la escala destino en español colombiano, los traductores difirieron en algunas palabras, como: posibilitar y permitir, asistencia y ayuda, encima de peso y sobrepeso, cuidado en casa y servicio domiciliario, dosis y trago; en general, hubo diferencias no significativas en cuanto a redacción, ya que no afectaban el entendimiento de la escala.

En la retro traducción se evidenció traducciones muy literales, pues algunos traductores incluso no tuvieron en cuenta el tiempo en el que se encontraba el verbo, como sucedió con la palabra “*parou”* y “*parada*”; sin embargo, en general ambos traductores mantuvieron el fin evaluativo de la escala, por lo cual, se dejaron aquellas palabras que más se adecuaban a la escala original. Finalmente, las síntesis fueron realizadas en consenso entre los investigadores, autores y traductores, tanto de la traducción inicial como de la retro-traducción, en donde se encontraron diferencias en algunos conceptos de los ítems, por lo tanto, se optó por hacer uso de sinónimos que se alienaban con el contexto cultural clínico, sin perder el alcance de la escala, buscando establecer una versión en español comprensible para Colombia.

### Validación de contenido por especialistas

El comité de especialistas estuvo conformado por 11 profesionales de salud en Colombia, se encontró que el mayor número de personas eran de sexo femenino, pertenecientes al departamento de Antioquia, seguido de Santander, con edad promedio de 40 ± 11.84 años. Igualmente, se evidenció que la formación en posgrados va orientada hacia la realización de cursos como: especialización, maestría y doctorado; que se presentan como único estudio académico en posgrado o de forma conjunto con varios estudios académicos en posgrado. La experiencia de los especialistas en su mayoría era superior a 10 años, y sus áreas laborales estaban centradas en la asistencia y docencia, ver Tabla 1.

**Tabla 1:** Caracterización sociodemográfica de los especialistas. 2021

Variable	n= 11
Sexo, Femenino!	10 (90.91)
Edad en años*	40 ± 11.84
Departamento de residencia	
Antioquia!	5 (45.45)
Córdoba!	2 (18.18)
Santander!	3 (27.27)
Rio Grande do Sul!	1 (9.09)
Formación en posgrad o	
PhD!	1 (9.09)
PhD (en curso)!	1 (9.09)
Especialista, PhD!	1 (9.09)
Especialista, Maestría, PhD!	1 (9.09)
Especialista, Maestría, PhD (en curso)!	3 (27.27)
Maestría!	2 (18.18)
Maestría (en curso)!	1 (9.09)
Maestría, PhD (en curso)!	1 (9.09)
Experiencia en Enfermería	
1 a 5 años!	1 (9.09)
6 a 10 años!	1 (9.09)
> 10 años!	9 (81.81)
Campo profesional	
Asistencial y docencia!	4 (36.36)
Asistencial, docencia y comunitaria!	1 (9.09)
Docencia!	5 (45.45)
Investigación!	1 (9.09)

*Media **±** desviación estándar; **f** n (%). PhD: Doctor.

Teniendo en cuenta los criterios de claridad del lenguaje, pertinencia práctica y relevancia teórica de los ítems, se consiguió el consenso de los especialistas en una ronda, estuvieron de acuerdo con las dimensiones de la escala, pero con relación a los ítems, realizaron algunas recomendaciones en cuanto a la claridad, las cuales fueron realizadas en la medida que se relacionarán con el objetivo de cada ítem, y el contexto cultural, manteniendo fidelidad a la estructura de la escala original.

Dentro de las recomendaciones, 5 especialistas sugirieron modificaciones en los ítems 4, 5 y 8 que hacen parte de los factores intrínsecos, al criterio de claridad, buscando que se adapten mejor al contexto cultural colombiano, estas modificaciones se fundamentaron desde la literatura científica, para hacer claridad sobre los conceptos a evaluar en cada ítem y su objetivo. La puntuación de estos ítems osciló entre 0.90 a 1.0, como se muestra en la Tabla 2.

**Tabla 2:** Evaluaciones de especialistas, por componentes de la versión en español de la escala RAC de evaluación de riesgo de infección de pacientes adultos hospitalizados.

			Evaluación por especialistas										
									Recomendaciones	n(%)			
			Claridad / Redacción		Pertinencia práctica		Relevancia teórica						
Dimensión	Número de ítem		SR	R	SR	R	SR	R		SR			R
	1	Género	x		x		x		-			-	-
	2	Edad	x		x		x		-	11	(100)	-	-
	3	Fumador	x		x		x		-	11	(100)	-	-
	4	Consumo de alcohol		x	x		x		Refinar la definición operativa	8	(72.7)	3	(27.3)
	5	Clasificación nutricional - IMC		x	x		x		Refinar la definición operativa	10	(90.9)	1	(9.1)
	6	Comorbilidades	x		x		x		-	11	(100)	-	-
	7	Lesión no quirúrgica	x		x		x		-	11	(100)	-	-
	8	Movilidad física		x	x		x		Se cambia definición por la cultura del medio	10	(90.9)	1	(9.1)
	9	Admisión previa	x		x		x		-	11	(100)	-	-
	10	Transferencia	x		x		x		-	11	(100)	-	-
	11	Unidad de admisión	x		x		x		-	11	(100)	-	-
	12	Duración de la estancia hospitalaria	x		x		x		-	11	(100)	-	-
		Cirugía durante el											
	13	ingreso actual o los 12	x		x		x		-	11	(100)	-	-
		meses anteriores											
	14	Procedimientos invasivos	x		x		x		-	11	(100)	-	-
Factores extrínsecos		Tratamiento previo											
	15	f armacológico y/o no	x		x		x		-	11	(100)	-	-
		farmacológico											

SR: Sin recomendaciones; R: Recomendaciones; IMC: Índice de Masa Corporal.

El comité de especialistas llegó al consenso de que la relevancia y pertinencia de los ítems tenían vínculo al contexto clínico, sin desconsiderar la finalidad de la escala, dando como resultado un IVC global de la escala de 0.98, como se señala en la Tabla 3.

**Tabla 3:** Análisis de la concordancia de la versión en español de la escala RAC de evaluación de riesgo de infección en pacientes adultos.

Dimensión	Número de ítem	Ítem	IVC			> 1 LU
			Claridad	Pertinencia	Relevancia	
Factores Intrínsecos	1	Género	1.00	1.00	1.00	
	2	Edad	1.00	0.90	0.90	
	3	Fumador	1.00	0.90	1.00	
	4	Consumo de alcohol	1.00	1.00	1.00	
	5	Clasificación nutricional - IMC	1.00	1.00	1.00	
	6	Comorbilidades	1.00	1.00	1.00	
	7	Lesión no quirúrgica	1.00	1.00	1.00	
	8	Movilidad física	0.90	0.90	0.90	
Factores Extrínsecos	9	Admisión previa	1.00	1.00	1.00	0.98
	10	Transferencia	1.00	1.00	1.00	
	11	Unidad de admisión	1.00	1.00	1.00	
	12	Duración de la estancia hospitalaria	1.00	1.00	1.00	
	13	Cirugía durante el ingreso actual o los 12 meses anteriore	s 1.00	1.00	1.00	
	14	Procedimientos invasivos	1.00	1.00	1.00	
	15	Tratamiento previo farmacológico y/o no farmacológico	1.00	1.00	1.00	
Proporción de especialistas/ Ítems - Índice de Validez de Contenido Total			0.99	0.98	0.98	

IVC: Índice de validez de contenido; E-IVC: Índice de validez de contenido de escala; IMC: Índice de Masa Corporal.

## Discusión

Este estudio mostró una adecuada AT y validez de contenido de la Escala RAC de Evaluación del Riesgo de Infección en el adulto hospitalizado para uso en Colombia. La escala RAC en su versión en español es comprensible y guarda coherencia con el instrumento original, de modo que logra sus objetivos evaluativos para la identificación de IAAS, lo que permite disminuir la brecha de desconocimiento existente en la práctica de enfermería en el entorno clínico.

En relación a estudios similares, se encontró la escala *St Thomas's risk assessment tool in falling elderly Inpatients* (STRATIFY)^28^^,^^29^, la cual fue diseñada en 1997 para valorar el riesgo de caídas en adultos mayores durante la hospitalización, cuyo idioma original es en inglés y estaba siendo usada con una traducción simple a español, sin haber sido previamente adaptada ni validada para el contexto sanitario de España, a pesar de las recomendaciones de la literatura que sugieren realizar este proceso a los instrumentos, antes de su utilización. Al momento de realizar la AT para el contexto de España en 2014, se presentaron desacuerdos en dos de las cinco preguntas que conforman el instrumento, siendo necesario incluir una oración explicativa para alcanzar la equivalencia conceptual, pero aún así se mantuvo la estructura, equivalencia semántica y la cantidad de ítems de la herramienta. Se tiene conocimiento que también fue adaptada en Australia, Bélgica, Canadá, Francia, Holanda e Italia^30^.

Asimismo, el interés por mantener la salud y seguridad del paciente a través de la detección y mitigación de los riesgos y peligros, conlleva al uso de cuestionarios como *Avaliagáo da Gestáo de Riscos Assistenciais em Servidos de Saúde* (AGRASS, por su siglas en portugués), que en su AT y validez de contenido requirió de modificaciones, en donde se validaron 39 ítems de los 40 ítems del instrumento original, y que se adaptaron al contexto cultural de Costa Rica, mediante el uso de sinónimos conservando el objetivo evaluativo del instrumento^31^, similar a los resultados de AT y validez de contenido al español de la escala RAC.

De lo anterior, se deriva la importancia de realizar la AT de cualquier instrumento antes de su uso en una cultura diferente a la de origen, debido a que una traducción literal de cualquier tipo de instrumento puede llevar a una apreciación equivoca y por ende a una invalidez de dicho instrumento. Es así como la AT y la validez de contenido cumplen un papel fundamental en el proceso de traducción, ya que se pueden evitar los errores de interpretación^32^. Durante todo el proceso de la AT y traducción del instrumento “Escala RAC de Evaluación del Riesgo de Infección en el Adulto Hospitalizado”, se siguieron todos las etapas metodológicas que se recomiendan en la literatura, teniendo siempre en cuenta el lenguaje local y sin realizar traducciones textuales en cada uno de los ítems^11^.

En cada una de las etapas, se siguió un riguroso proceso metodológico con el fin que el entendimiento de la escala y sus puntuaciones continuaran siendo equivalentes, tanto en la escala original como la versión en español de Colombia. En este mismo sentido, la realización de la validación de contenido posibilitó que se pudieran verificar que cada uno de los ítems que conforman la escala fueran claros en cuanto a su redacción, contaran con una pertinencia práctica y tuvieran una relevancia teórica, buscando siempre alcanzar el objetivo de la escala. En algunos casos fue necesario modificar, agregar o suprimir palabras para asegurar la exactitud y calidad de la información. Ambas dimensiones tanto intrínseca como extrínseca, contaron con índices de validez de contenido relevantes con un E-IVC de 0.98, valor superior con relación al E-IVC de la escala original en portugués de 0.90^18^, lo cual confirma que la escala cumple su objetivo de medir la identificación de los riesgos presentes para adquirir una IAAS en adultos hospitalizados.

La necesidad desde la práctica de contar con un instrumento válido que permita medir este fenómeno es indispensable en la seguridad del paciente^33^, ya que permite abordar todas las esferas del ser humano, así como la mejora en el cuidado brindado por los profesionales de la salud, al centrar la atención en las necesidades principales del paciente, logrando de esta forma desarrollar un plan de cuidados mucho más eficiente.

La AT y validez de contenido de la versión en español de la escala RAC genera un gran impacto en la atención en salud del adulto hospitalizado y como herramienta útil para el personal de la salud, dado que contribuye a prestar un mejor cuidado a los pacientes desde su ingreso a la entidad de salud^16^. Por lo cual, se sugiere usar la escala RAC en los diversos ambientes hospitalarios de Colombia, con el fin de socializar diferencias en cuanto a la comprensión de las dimensiones y los ítems que conforman la escala, pero también se hace necesario realizar nuevas investigaciones con el fin de identificar otras formas igualmente predictivas, para la identificación de otros riesgos y así construir una sólida cultura de seguridad del paciente.

Una de las fortalezas del estudio es que el comité de especialistas estuvo conformado por profesionales de varias regiones de Colombia y con ampliada experiencia clínica y de formación de posgrado. No obstante, una posible limitación de este estudio es que el comité de especialistas estuvo compuesto solo por profesionales de enfermería, razón por la cual es recomendable que la validación por consenso de especialistas también pueda ser realizada por otros profesionales de la salud, dado que se espera que la escala RAC sea usada por cualquier miembro del personal sanitario, teniendo en cuenta que la atención hospitalaria requiere de la intervención de un equipo multidisciplinar.

## Conclusiones

Los hallazgos de este estudio soportan la AT y validación de contenido de la escala RAC para su uso en Colombia. Futuros estudios que usen esta escala pueden orientar a una mejor comprensión del riesgo de IAAS y ayudar a los profesionales de la salud en la toma de decisiones para implementar intervenciones que favorezcan la seguridad del paciente.

La Escala RAC de Evaluación del Riesgo de Infección en el adulto hospitalizado, es una herramienta accesible y de bajo costo para la implementación en las diferentes instituciones de salud, que busca priorizar la identificación de factores de riesgo para IAAS, con la posterior planeación y ejecución de intervenciones oportunas e individualizadas, que promuevan la integridad y seguridad del paciente. Además, la escala RAC en su versión en español para uso en Colombia puede favorecer la gestión de riesgos y el control de infecciones hospitalarias para lograr mejores prácticas de cuidado y mejores indicadores de calidad asistencial.
